# Using Mobile Technology to Provide Personalized Reminiscence for People Living With Dementia and Their Carers: Appraisal of Outcomes From a Quasi-Experimental Study

**DOI:** 10.2196/mental.9684

**Published:** 2018-09-11

**Authors:** Elizabeth A Laird, Assumpta Ryan, Claire McCauley, Raymond B Bond, Maurice D Mulvenna, Kevin J Curran, Brendan Bunting, Finola Ferry, Aideen Gibson

**Affiliations:** 1 Institute of Nursing and Health Research Ulster University Derry United Kingdom; 2 School of Computing Ulster University Belfast United Kingdom; 3 School of Computing, Engineering & Intelligent Systems Ulster University Derry United Kingdom; 4 Psychology Research Institute Ulster University Derry United Kingdom

**Keywords:** dementia, evaluation, mobile apps, reminiscence, research, technology, mobile phone

## Abstract

**Background:**

Dementia is an international research priority. Reminiscence is an intervention that prompts memories and has been widely used as a therapeutic approach for people living with dementia. We developed a novel iPad app to support home-based personalized reminiscence. It is crucial that technology-enabled reminiscence interventions are appraised.

**Objective:**

We sought to measure the effect of technology-enabled reminiscence on mutuality (defined as the level of “closeness” between an adult living with dementia and their carer), quality of carer and patient relationship, and subjective well-being.

**Methods:**

A 19-week personalized reminiscence intervention facilitated by a program of training and a bespoke iPad app was delivered to people living with dementia and their family carers at their own homes. Participants (N=60) were recruited in dyads from a cognitive rehabilitation team affiliated with a large UK health care organization. Each dyad comprised a person living with early to moderate dementia and his or her family carer. Outcome measurement data were collected at baseline, midpoint, and intervention closure.

**Results:**

Participants living with dementia attained statistically significant increases in mutuality, quality of carer and patient relationship, and subjective well-being (*P*<.001 for all 3) from baseline to endpoint. Carers attained nonsignificant increases in mutuality and quality of carer and patient relationship and a nonsignificant decrease in subjective well-being.

**Conclusions:**

Our results indicate that individual-specific reminiscence supported by an iPad app may be efficient in the context of early to moderate dementia. A robust randomized controlled trial of technology-enabled personalized reminiscence is warranted.

## Introduction

### Background

Dementia is an umbrella term that encompasses at least 40 conditions that feature progressive cognitive decline and are more prevalent in older age. In tandem with international aging demographics, the prevalence of dementia and associated costs have risen substantially. The estimated annual UK cost of dementia is over £26 billion [1], and this is higher than the combined costs for cancer, stroke, and heart disease. There is increasing evidence that nonpharmacological interventions for the symptoms of dementia can have commensurate effectiveness to pharmacological treatment and may be preferable where medication can cause negative side effects [2-4]. The progressive nature of dementia presents a challenge for families providing care to a relative with this condition [5-7]. Not surprisingly, the World Health Organization (WHO) has prioritized dementia as a global public health concern and has recommended that more research should be undertaken to inform supportive interventions for people living with dementia and their families [8].

Reminiscence refers to a range of psychosocial interventions that prompt memories and has been widely used as a therapeutic approach for people living with dementia and their carers [9,10]. Technology-based reminiscence increases opportunities to participate in conversations and enhances the social interactions of people living with dementia and their carers [10]; furthermore, it enables remote reminiscence to be delivered at home [11]. Traditional reminiscence utilizes collections of resources such as memory boxes that can stimulate a range of senses, including touch, taste, and smell. In contrast, technology-based reminiscence is reliant on visual and auditory memory prompts. These limitations may be offset by the portability, mobility, and utility of technology-based reminiscence systems to deliver personalized reminiscence experiences.

A systematic review [12] of technology-supported reminiscence therapy identified 44 papers that met the selection criteria. Although limited by the small sample sizes of some of the studies, the authors concluded that there were benefits to using information and communication technology (ICT) for reminiscence interventions. These benefits include access to rich and engaging multimedia reminiscence materials [13,14], opportunities for people living with dementia to participate in social interactions and take ownership of conversations [15,16], and a reduction in motor deficit-related barriers when interacting with media [16,17]. In the abovementioned review [12], 10 reviewed papers reported on the use of “reminiscence kits” that featured a technological component. Audio was a major component of these reminiscence kits, but impact evaluation was not reported. One study [17] examined the attitudes of older people (N=19) toward using an iPad to aid reminiscence. Participants in the study were randomly allocated to reminisce using either an iPad or more traditional images and cards. The results from that study indicated that participants enjoyed using the iPad. In a follow-up mixed methods design, a mobile app called “Memory Matters” was developed to promote reminiscence [15]; 18 people living with dementia and 8 family carers were asked to use MM for a period of 4 weeks. Consistent with the findings of a more recent study that explored a similar device [18], the technology-supported reminiscence was favorably evaluated. Family carers enjoyed discussing the early years with their relative, and on several occasions, the people living with dementia shared memories in a direct response to prompts provided by MM. People living with dementia who had only interacted minimally, or who had never spoken before, were observed to interact and support each other while using the app. These findings support the social engagement potential of mobile devices in the context of family caregiving in dementia [19,20].

As dementia progresses, it is common for carers to report a “disappearance of the relationship” [21,22]. There is a need to support caregiving relationships in order to protect mental and physical well-being of the carers [23,24]. ICT has an important role to play in this endeavor by supporting social connectivity [25]. It is important, therefore, that in studies of technology-based reminiscence, family carers are included in addition to people living with dementia.

Our research team was motivated by the rising acceptability of health apps to develop and test the feasibility of a novel app to deliver personalized reminiscence among people living with dementia and their carers. Consistent with recommendations [12], validated and standard outcome measures were selected for the appraisal of efficacy. Mutuality is a scale that measures closeness in a relationship [26]; WHO-Five Well-Being Index (WHO-5) is a short scale for measuring emotional well-being [27], and Quality of Carer-Patient Relationship scale (QCPR) is a scale of family caregiving [28]. All 3 scales have been tested in dementia research, but not in reminiscence research. This paper contributes to the evidence base by reporting the preliminary efficacy of technology-based personalized reminiscence facilitated by a program of training and an iPad app on mutuality, quality of caregiving relationships, and subjective well-being among people living with dementia and their family carers.

### Development of a Reminiscence App

The size, capacity, and low cost of ubiquitous mobile devices have made them an attractive option for technology-based reminiscence systems. As part of this study, a cross-platform device agnostic tablet app (called InspireD: an acronym for Individual-Specific Reminiscence for People living with Dementia) was developed to facilitate reminiscing activity. The two primary aims of the app were to enable people living with dementia and their family carers to select and store personalized memorabilia (photographs, videos, sounds, music, etc) and to provide easy access to these visual and audiovisual cues to support bespoke reminiscence.

**Figure 1:**
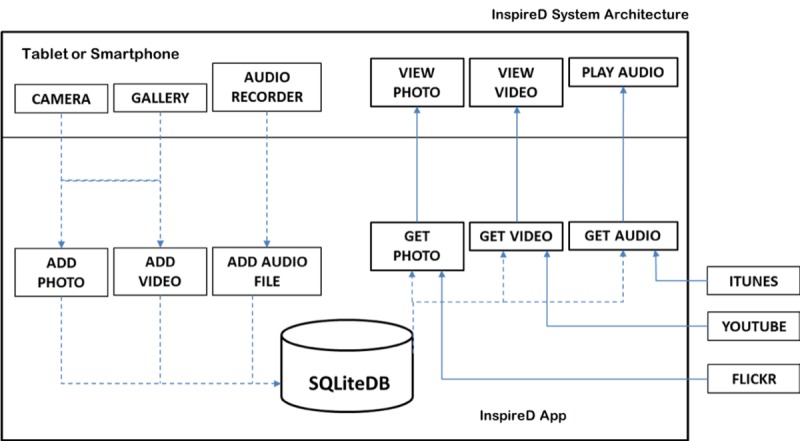
InspireD app system architecture SQLiteDB SQLite database.

The InspireD app was developed [29] with input from the Reminiscence Network Northern Ireland and a user development group that comprised a total of 7 dyads, with each dyad comprising a person living with dementia and his or her primary caregiver (n=14). The Agile software development approach [30] was adopted to allow the functional prototype to be created early in the development lifecycle, with testing for usability and refinement taking place throughout the development process [29]. The app was implemented using Appcelerator Studio (Appcelerator Titanium SDK, US), an Eclipse-based integrated development environment that provides an environment to build, test, package, and publish apps for various platforms, including iOS and Android. The code is written in JavaScript, with native user interface (UI) elements being invoked at runtime. It incorporates local facilities for persistent data storage in SQLite database and facilitates the use of third-party app programming interfaces for Flickr and YouTube (Figure 1).

**Figure 2:**
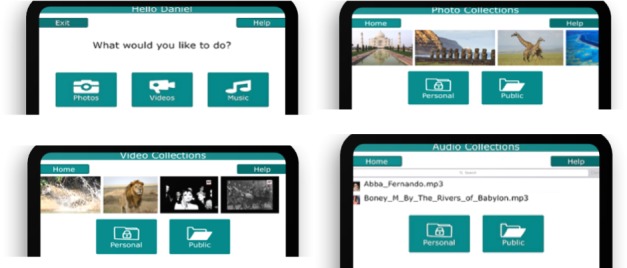
The InspireD reminiscence app user interface.

The app consists of a UI that is usable and responsive across a variety of mobile devices (tablets, mobile phones). It is also possible to use the system on a personal computer or laptop via the Web browser. The main user (and co-users, ie, carers) can upload images, videos clips, and audio clips to the app. SQLite database functionality is used to store and manage data natively. The main UI consists of a simple screen for people living with dementia to upload files with help from a reminiscence trainer or a family caregiver. A multiscreen layout allows users to choose which memorabilia they wish to access: view photos, watch videos, listen to audio files, and browse selected resources (Figure 2).

The design is minimalist, using verbal descriptors as well as images and icons to reinforce and indicate functionality to the user. Data are organized and presented primarily in the form of on-screen menus. The welcome screen is a simple log-in screen where the users confirm their identity by clicking a photo of themselves. The user data are contained within a local SQLite database, which can be easily queried with the reporting services enabled. Multimedia reminiscing resources (photos, videos, and audios) are also stored locally in the app data directory.

The InspireD app incorporates a logging facility for 5 canonical events. These are entry (logging in), admin (adding a photo, deleting an audio, etc), reminiscing (viewing a video, viewing a photo, etc), ecological momentary assessment (EMA) questions, and exit (logging out). Usage data across the course of the intervention were collected via secure email and statistically analyzed, and the findings have been published [31]. EMA is influenced by Kurt Lewin [32]. The use of “in-the-moment” approaches, along with rigorous measurement techniques in psychometric research, has been validated in recent research [33-36]. In our study, EMA involved the delivery of a small series of 5 questions directly to participants through the app; the feasibility of this approach in the context of dementia care is being appraised and will be reported in a subsequent paper. The InspireD system was designed with scalability in mind for future enhancements as it is envisaged that the final version will be a secure, cloud-based app accessible via a secure internet connection for authorized users. While the design incorporated the ability to store content locally on the device or to upload it to a cloud-based storage, a decision was made by the team that for the feasibility study, all content would be stored locally on the device.

## Methods

### Design

This paper reports on a feasibility study that incorporated a quasi-experimental design. An intervention of home-based personalized reminiscence, supported by a program of training and a novel iPad app, was examined for preliminary efficacy using 3 outcome measures pertaining to mutuality, emotional well-being, and quality of carer-patient relationships. The quasi-experimental design is appropriate and a common design for assessing the feasibility of novel technological interventions [37-39]. In line with quality standards, repeated measures testing was employed. Data were collected at baseline, midpoint, and intervention close. Table 1 outlines the intervention activities and data collection time-points.

The model of reminiscence that was utilized to underpin the training intervention was that of simple reminiscence [40], which encompasses mainly unstructured autobiographic storytelling and triggers that generate spontaneous reminiscence, often within a relational context, such as special days or events shared by friends and family. The goal of this approach is to enhance social contacts and short-term well-being while also supporting intergenerational bonding [40,41]. Our intervention was designed to cater for the needs, preferences, and interests of people living with dementia and their family carers. As a measure of quality, the reporting of this study adheres to the Transparent Reporting of Evaluations with Nonrandomized Designs (TREND) statement [42].

### Settings

The setting was a large health and social care trust in a region of the United Kingdom. The trust catchment area is a mix of rural and urban communities serving a population of approximately 300,000 people, with an estimated 2717 of them living with dementia. Recruitment was facilitated by the trust’s community mental health team for older people and through the trust’s cognitive rehabilitation team as engagement with the latter was indicative of a diagnosis of early to moderate dementia.

### Participants

A purposive sampling strategy was used to recruit 30 caregiving dyads (30 persons living with dementia and 30 carers). A sample size of 40-50 is recommended as sufficient for a feasibility study to estimate the total sample size across parameters to inform a future randomized controlled trial (RCT) [43]. Our participants were predominantly older people, and there was potential for significant dropout. With that in mind, we increased our sample size to 60.

Inclusion and exclusion criteria were developed to minimize the potential for bias in the recruitment process. We included people who (1) had a diagnosis of early to moderate dementia, (2) were able to communicate and understand conversations, and (3) were aware of their dementia diagnosis; furthermore, we included family carers who were (1) aged ≥18 years, (2) caring for a family member living with dementia meeting the above criteria (either cohabiting or non-cohabiting), and (3) aware of their relative’s dementia diagnosis. Individuals with a major illness or disability that hindered their ability to engage in the study were excluded. Recruitment was commenced in April 2016 and continued until the sample size of 30 dyads (N=60) was achieved in October 2016.

Ethical considerations principally pertained to voluntariness, supporting separate informed consent for the people living with dementia and their carers, handling and storage of data, and right to withdraw from the study. The study received ethical approval (REC Ref 16/NI/0002) in line with regional and National Health Service Trust research governance.

### Primary Outcome Measure

The primary outcome measure was mutuality, defined as the positive quality of the relationship between the carer and the care recipient [26]. The Mutuality scale consists of 15 items. A sample item includes “How attached are you to him or her?” A 5-point scale is used, ranging from 0 (not at all) to 4 (a great deal). Higher scores indicate a higher level of mutuality, which may support relationships in difficult circumstances. The Mutuality scale has been tested for validity in previous studies of family caregiving and has demonstrated internal consistency [44,45]. The mean was calculated across the response scores for data analysis [45,46].

### Secondary Outcome Measures

The secondary outcome measures comprised QCPR [28] and WHO-5 [27,47]. The QCPR is a 14-item scale measuring relationship quality, including level of warmth and level of criticism. The scale has demonstrated good internal consistency and concurrent validity with other measures of relationship quality and carer stress [48]. Responses are rated using a 5-point Likert scale, scored from 1 (totally disagree) to 5 (totally agree). The 6 items measuring criticism and conflict are reverse scored in computation [28]. Total QCPR scores are utilized for data analysis. A total score >42 is indicative of a good relationship.

The WHO-5 comprises 5 questions that tap into the subjective well-being of participants; it has been extensively tested for validity [49,50] and reliability [51,52] scale items are scored from 0-5 and then totaled, giving a potential raw score ranging from 0 to 25. It is recommended that in studies assessing change over time, the WHO-5 raw scores are transformed to percentage scores for data analysis [53]. A total percentage score of ≤50 is an indication of low mood, and a score of ≤28 suggests likely depression, warranting further assessment.

### Intervention and Follow-Up

Participating dyads were provided with a new touch screen tablet device that hosted the novel InspireD reminiscence app. A package of 5 reminiscence training sessions influenced by evidence-informed guidelines [40,41] was delivered by a reminiscence trainer employed by the Reminiscence Network Northern Ireland. Three information technology (IT) training sessions were then provided by an IT assistant to support the participants in uploading their personal memorabilia and to use the reminiscence app independently. The reminiscence and IT training packages were provided face-to-face, at the homes of participants living with dementia. The estimated cost of the intervention, which included the training package together with the cost of the InspireD system, was £2570 per dyad. After training was completed, participants were requested to engage in simple reminiscing through the app 3 days per week for the following 12 weeks. Compliance was supported by a user-friendly instruction booklet. A phone number for the IT trainer was provided, should any technological issues arise.

Participants were followed up for a period of 19 weeks from baseline (T_0_), as outlined in Table 1. Midpoint measurement data (T_1_) were collected on week 13 from baseline, which was 6 weeks into the independent use of the reminiscence technology. Endpoint measurement data were collected on week 19 at closure of the intervention (T_2_). The data collection period was May 2016 to February 2017. Given the study design, there was no control group, and given the nature of the intervention, it was not possible to blind the participants or the trainers administering the intervention. All data were uploaded to IBM SPSS version 23 [54] using unique anonymized identification codes by the research assistant. The researcher responsible for analyzing the data and interpreting the results used that anonymized dataset.

### Statistical Analysis

We used descriptive statistics to describe and synthesize the data pertaining to the characteristics of the participants. Missing data analysis, as recommended, was undertaken to discern possible patterns and challenges in the selected measurement tools [55]. We performed chi-square tests for analysis of nominal variables and independent *t* tests to compare measurement scores in Mutuality, QCPR, and WHO-5 across the dyad relationship (people living with dementia and their carers) and across gender at baseline; chi-square tests were not performed when numbers in categories were less than 5. Paired *t* tests were performed to investigate the differences in scores across 2 time-points. Furthermore, we performed within and between repeated measures analysis of variance (ANOVA) to investigate the impact of the intervention over time.

**Table 1:** Intervention activities and data collection time-points.

Timescale and activities				Data collection time-points		Repeated measures
**Preintervention**						
	Baseline		Baseline (T_0_)		Mutuality WHO-5^b^ QCPR^c^	
**19-week intervention**						
	**Weeks 1-6 training**					
		Reminiscence training package (5 sessions)	N/A^a^		N/A	
		Information technology training package (3 sessions)	N/A		N/A	
	**Week 7-19 reminiscence using iPad app**					
		Home reminiscence begins in week 7	N/A		N/A	
		Home reminiscence continues in week 13	Midpoint (T_1_)		Mutuality WHO-5 QCPR	
		Home reminiscence ends in week 19	Endpoint (T_2_)		Mutuality WHO-5 QCPR	

^a^N/A: not applicable.

^b^WHO-5: World Health Organization–Five Well-Being Index.

^c^QCPR: Quality of the Carer and Patient Relationship scale.

Correlational tests were performed to investigate relationships between continuous variables. On an intention-to-treat basis [55,56], missing data for Mutuality, QCPR, and WHO-5 were managed using the expectation-maximization imputation approach.

## Results

### Baseline Assessment

A baseline assessment of demographic details, Mutuality, QCPR, and WHO-5 was conducted prior to the program of reminiscence and IT training. We recruited 60 participants, in 30 dyads, in this study. Of them, a total of 58 participants (29 dyads) were retained in the study at completion. The participant characteristics and baseline measurement scores are presented in Table 2. Of all the participants with dementia, 67% (20/30) were men. A chi-square test for independence (with Yates continuity correction) was performed, and it revealed that the gender composition of the carer participants was different from that of participants living with dementia. Of all the carers, 80% (24/30) were women (*P*=.001). The age range of participants living with dementia was 61-94 years and that of the carers was 31-91 years. An independent *t* test revealed that the age of the carers (mean 67 years, SD 14.8) was significantly lower than that of the participants living with dementia (mean 79 years, SD 8.9; *P*<.001). In 23 of the dyads, the carer was living in the same house as the participant living with dementia. The majority of the participants living with dementia had little or no IT experience, whereas the majority of carer participants had at least moderate IT experience.

There were no missing data at baseline. Mean mutuality score at baseline was 3.13 (SD 0.68), indicating a moderate level of closeness in the relationship. Visual inspection of the histogram and Q-Q plots indicated a reasonable but positively skewed distribution.

**Table 2:** Baseline characteristics of participants.

Characteristic		Number of participants (N=60)	People living with dementia (n=30)	Family carers (n=30)	*P* value^a^
Age (years), mean (SD)		73 (13)	79 (8.9)	67 (14.8)	<.001
Age range (years)		31-94	61-94	31-91	N/A^b^
**Gender, n (%)**					
	Male	26 (43)	20 (67)	6 (20)	N/A
	Female	34 (57)	10 (33)	24 (80)	.001
**Marital status, n (%)**					
	Married	47 (78)	22 (73)	25 (83)	N/A
	Widowed	9 (15)	8 (27)	1 (3)	N/A
	Separated or single	4 (6.7)	0 (0)	4 (13)	N/A
**IT^c^ experience, n (%)**					
	Little or none	35 (58)	24 (80)	11 (37)	N/A
	Moderate	21 (35)	5 (17)	16 (53)	N/A
	A lot	4 (7)	1 (3)	3 (10)	N/A
Home internet access, n (%)		52 (87)	25 (83)	27 (90)	N/A
**Hobby choices, n (%)**					
	Social	29 (48)	14 (47)	15 (50)	N/A
	Physical fitness	19 (32)	8 (27)	11 (37)	N/A
	Creative	7 (12)	4 (13)	3 (10)	N/A
	No hobby	5 (8)	4 (13)	1 (3)	N/A
**Repeated measures, mean (SD)**					
	Mutuality	3.13 (0.68)	3.24 (0.54)	3.02 (0.79)	.22
	QCPR^d^	57.4 (7.9)	58.1 (7.1)	56.7 (8.6)	.52
	WHO-5^e^	61.0 (23.9)	60.8 (26.2)	61.2 (21.8)	.94

^a^*P* values <.05 indicate significance.

^b^N/A: not applicable.

^c^IT: information technology.

^d^QCPR: Quality of Carer-Patient Relationship scale.

^e^WHO-5: World Health Organization–Five Well-Being Index.

There was no significant difference in baseline mutuality for participants living with dementia and carers (*P*=.22). There was a statistically significant difference between mutuality for men (mean 2.9, SD 0.78) and women (mean 3.3, SD 0.56), with women having higher scores (95% CI −0.756 to −0.026; *P*=.04). No relationship was discerned between age and baseline mutuality (r=0.09).

Mean QCPR score was 57.4 (SD 0.9), indicating a good relationship. A Kolmogorov-Smirnov statistic of 0.057 was suggestive of a normal distribution, supported by visual inspection of histograms and Q-Q plots. There was no significant difference in baseline QCPR score between participants living with dementia and carers (*P*=.52) or between men and women (*P*=.91). There was a positive but weak correlation between age and QCPR (r=−0.123), which failed to reach statistical significance (*P*=.35).

Mean WHO-5 score was 61.0 (SD 23.9), indicating a moderate level of subjective well-being. The Shapiro-Wilks statistic (appropriate for a small sample) of 0.052 suggested normality, and this was supported by visual inspection of histograms and Q-Q plots. There was no significant difference in WHO-5 score between participants living with dementia and carers (*P*=.94) or between men and women (*P*=.40). No relationship was discerned between age and baseline WHO-5 (r=−0.04).

### Missing Data Analysis

At T_1_ (midpoint), 5% (3/60) participants had missing data: participant 50 had 2.9% missing data; participant 11 had 5.9% missing data, and participant 44 was unavailable for data collection due to a hospital admission. At T_2_ (endpoint), 6.6% (4/60) participants had missing data: participant 20 had 2.9% missing data; participant 43 was unavailable due to a hospital admission, and one participant had died and her carer withdrew from the study.

### Intention-to-Treat Analysis

Paired-samples *t* tests were conducted to compare baseline and endpoint measurement scores. There was a statistically significant increase in mutuality scores of participants living with dementia from baseline (mean 3.24, SD 0.545) to endpoint (mean 3.64, SD 0.274; 95% CI −0.56 to −0.23; *P*<.001, two-tailed). Furthermore, there was a statistically significant increase in QCPR scores of participants living with dementia from baseline (mean 58.07, SD 7.12) to endpoint (mean 63.2, SD 4.32; 95% CI −7.42 to −2.84; *P*<.001, two-tailed). Similarly, there was a statistically significant increase in WHO-5 scores of participants living with dementia from baseline (mean 60.8, SD 26.2) to endpoint (mean 70.6, SD 21.4; 95% CI −14.8 to −4.84; *P*<.001, two-tailed).

Regarding the carers, there was an increase in mutuality from baseline (mean 3.02, SD 0.79) to endpoint (mean 3.07, SD 0.60), but the increase was not statistically significant (*P*=.52). Similarly, an increase in carer QCPR scores from baseline (mean 56.7, SD 8.66) to endpoint (mean 57.9, SD 8.26) was not statistically significant (*P*=.28). There was a decrease in carer WHO-5 scores from baseline (mean 61.2, SD 21.8) to endpoint (mean 60.2, SD 23.4), but the change in the scores was not statistically significant (*P*=.74).

Mixed between-within subjects ANOVA was performed to assess the impact of the reminiscence intervention over time and between participants living with dementia and the carers. For the participants living with dementia, mean mutuality increased from baseline to midpoint and then further increased at endpoint. For the carers, mean mutuality peaked at midpoint. A similar pattern for participants living with dementia and carers was observed in mean QCPR scores over time. Regarding WHO-5, the mean scores of participants living with dementia increased from baseline to midpoint, with a further increase at endpoint. For the carers, mean WHO-5 scores decreased from baseline to midpoint and then increased to the end point. Mean outcome measurement scores, SDs, time-related *P* values, and pattern difference *P* values are presented in Table 3.

A statistically significant effect of the intervention on mutuality was demonstrated over time, Wilks lambda=0.77, F(2,57)=8.17, *P*=.001, partial eta squared=0.22. The pattern of mutuality scores for the participants living with dementia and the carers was significantly different, Wilks lambda=0.87, F(2,57)=4.23, *P*=.02, partial eta squared=0.129, with participants living with dementia exhibiting higher scores. A statistically significant effect of the intervention on QCPR was demonstrated over time, Wilks lambda=0.777, F(2,57)=8.15, *P*=.001, partial eta squared=.223. The pattern of QCPR scores for participants living with dementia and carers was statistically significant, Wilks lambda=0.88, F(2,57)=3.72, *P*=.03, partial eta squared=0.116. The participants living with dementia exhibited higher scores. Overall, the intervention did not demonstrate a significant effect on WHO-5 scores over time, Wilks lambda=0.90, F(2,57)=2.94, *P*=.06, partial eta squared=0.09. However, a statistically significant difference was found in the pattern of scores between the participants living with dementia and the carers, Wilks lambda=0.85, F(2,57)=4.90, *P*=.01, partial eta squared= 0.147. The participants living with dementia exhibited a pattern of higher scores.

### Estimation of Sample Size for a Future Randomized Controlled Trial

A linear mixed model for a 2-way repeated measures ANOVA (fixed effects) was used to analyze the data. The between effect was dyad role, that is, participants living with dementia versus carers. The statistical power for the between effect in the model, based on the results from the mutuality measure, was 36 individuals per group (total=72). The power to detect the effects was set at 0.9 in all of the analyses.

For the within effect (repeated measures for both carers and those living with dementia), to detect the main effect of time (within subject effect), a sample of 16 respondents would be required in each group (total=32). For between-within subjects (interaction), to detect the interaction of condition (carer vs those living with dementia) and time, a sample of 39 individuals in each condition (total=78) would be required to detect an effect similar to that present in the previous study, with a statistical power of 0.9.

**Table 3:** Measures across time.

Measure and participant		Baseline (T_0_), mean (SD)	Midpoint (T_1_), mean (SD)	Endpoint, (T_2_), mean (SD)	N	Time *P* value	Pattern *P* value
**Mutuality**		3.1 (0.687)	3.4 (0.55)	3.4 (0.543)	60	.001	N/A
	PLWD^a^	3.2 (0.556)	3.6 (0.203)	3.6 (0.275)	30	N/A^b^	N/A
	Carer	3.0 (0.799)	3.1 (0.67)	3.1 (0.601)	30	N/A	.02
	**QCPR^c^**							
	PLWD	58.1 (7.1)	61.3 (5.2)	63.2 (4.3)	30	N/A	N/A
	Carer	56.8 (8.7)	58.6 (7.4)	58.9 (8.3)	30	N/A	.03
	Total	57.4 (7.9)	59.9 (6.5)	60.6 (7.0)	60	.001	N/A
**WHO-5^d^**							
	PLWD	60.8 (26)	69.9 (18)	70.7 (21)	30	N/A	N/A
	Carer	61.2 (22)	56.5 (27)	60.3 (23)	30	N/A	.01
	Total	61.0 (24)	63.2 (24)	65.5 (23)	60	.06	N/A

^a^PLWD: person living with dementia.

^b^N/A: not applicable.

^c^QCPR: Quality of Carer-Patient Relationship scale.

^d^WHO-5: World Health Organization–Five Well-Being Index.

## Discussion

### Principal Findings

In this quasi-experimental study, we sought to appraise outcomes from a feasibility study of individual-specific reminiscence facilitated by a program of training and an iPad app. A total of 58 participants (29 dyads) were retained in the study at completion, supporting the understanding that neither age nor a diagnosis of dementia are barriers to engagement in home-based research using technology. The main findings from the study are as follows: (1) statistically significant increases in mutuality, quality of carer and patient relationship, and emotional well-being of participants living with dementia from baseline to endpoint; (2) nonsignificant increases in mutuality and QCPR and a nonsignificant decrease in WHO-5 scores for the carer participants from baseline to endpoint; and (3) statistically significant differences in patterns of intervention effect across time, with the participants living with dementia exhibiting patterns of higher scores. It is difficult to determine the clinical significance of these changes as this was outside the remit of this feasibility study. However, a future RCT could include additional scales such as the mini-mental state examination [57] and Geriatric Depression Scale Short Form [58] in repeated measures testing.

### Comparison With Prior Work

There is increasing use of mobile computer software and rising acceptability of health promotion apps internationally [29,59-62]. It was foreseen that the future would bring opportunities for reminiscing facilitated by touch screen interfaces [10]. Ours was a feasibility study with the objectives of testing a novel intervention of individual-specific reminiscence and investigating its impact. The total number of participants (N=60) recruited to this study is a significant increase from previous technology-enabled reminiscence studies [12]. The 3 outcome measures utilized in our study were mutuality [26], Quality of the Carer and Patient Relationship [28], and WHO-5 [47]. A strength of our study is that all 3 tools have previously undergone extensive testing for validity and reliability and were sufficiently sensitive to deliver statistically significant results. Our reminiscence intervention differed in a number of ways from the approaches taken in recent studies [12]: (1) our intervention was home based; (2) the participating dyads received a program of individual-specific training in reminiscing and IT, and (3) the reminiscing activity was supported by an iPad app hosted on tablet software with each of the participants having his or her own unique access log-in details. Our findings suggest that technology-based reminiscence may be able to support mutuality and quality of informal caregiving relationships, in contrast with negative trends observed in longitudinal studies among caregiving dyads [22,44]. We cannot make direct comparisons between our results and those of other technology-based reminiscence research due to the lack of appraisal of outcome in previous research. Our findings, however, add to the emerging evidence that technology-based reminiscence offers benefits in the context of family caregiving in dementia [12,15,18].

It is acknowledged internationally that family carers are the most important practical, personal, and economic supports for people with dementia [7,8] and that enduring caregiving roles in the context of dementia are associated with significant negative trends in mutuality and quality of life among family carers [22]. It would, therefore, not have been surprising if we had found significant negative trends in the outcome scores of carers over the course of our study. Our decision to deliver a home-based intervention was informed by research that suggested that research participation can pose a significant challenge to carers of people living with dementia [48]. Our research findings suggest that it is possible that the home-based nature of our intervention contributed to the statistically significant enhancements for participants with dementia, with no significant detriment to carers. To what extent carers would continue to support the intervention in a longer-duration study is worthy of consideration. It may be possible to develop the iPad app further to utilize a coaching companion to prompt, incentivize, and reward the carer. Taken together, our findings support the need for a robust RCT of home-based app-enabled personalized reminiscence. A stratified sampling strategy guided by mini-mental state examination scores, with matched controls, and a longer follow-up time of up to 2 years would address the unknown issue of how long the intervention effect might last.

### Limitations

This was a feasibility study, and it was important to maximize the exposure to the novel technology-based personalized reminiscence intervention. There are acknowledged challenges in the recruitment of people living with dementia and their family carers, and use of a comparison group would have reduced exposure to the intervention. Quasi-experimental designs, such as the one we adopted, cannot establish cause-and-effect relationships with certainty, but they can establish strong links. We cannot rule out a Hawthorne effect given the trial design and the possibility that pre-existing factors have influenced the results. Conclusions, therefore, have to be interpreted with caution. An additional limitation is the underrepresentation of women among the participants living with dementia in our study, given that women have been constituted as a marginalized majority in UK prevalence of dementia [63].

### Conclusions

Reminiscence has been promoted internationally as a means of enhancing standards of care and quality of life of people living with dementia and their family carers. Our study comprised a novel intervention of home-based reminiscence with repeated measures testing. The findings of this study indicate statistically significant enhancements in mutuality, quality of relationship, and subjective well-being for the participants living with dementia and nonsignificant enhancements in mutuality and quality of relationship for carers. These findings support an emerging body of evidence that purports that individual-specific psychosocial interventions have efficacy in the context of dementia. It is important to highlight that our study is not without limitations and that pre-existing factors may have influenced the results. Nonetheless, our intervention, comprising a program of training and use of a novel iPad app, may contribute to the ongoing development of home-based reminiscence in the context of dementia. Future research must be cognizant of the potential for women living with dementia to be underrepresented among participants and the importance of controlling pre-existing factors. A robust RCT of personalized reminiscence is worthy of consideration.

## Abbreviations

ANOVA: analysis of variance
EMA: ecological momentary assessment
ICT: information and communication technology
InspireD: Individual-Specific Reminiscence for People living with Dementia
IT: information technology
QCPR: Quality of Carer-Patient Relationship scale
RCT: randomized controlled trial
UI: user interface
WHO: World Health Organization
WHO-5: World Health Organization–Five Well-Being Index

## References

[1] Knapp M, Guerchet M, McCrone P, Prina M, Comas-Herrera A, Wittenberg R, Adelaja B, Hu B, King D, Rehill A, Salimkumar D, Prince M (2014). Alzheimer's Society.

[2] Lawrence V, Fossey J, Ballard C, Moniz-Cook E, Murray J (2012). Improving quality of life for people with dementia in care homes: making psychosocial interventions work. Br J Psychiatry.

[3] Gonzalez J, Mayordomo T, Torres M, Sales A, Meléndez JC (2015). Reminiscence and dementia: a therapeutic intervention. Int Psychogeriatr.

[4] Woods RT, Bruce E, Edwards RT, Elvish R, Hoare Z, Hounsome B, Keady J, Moniz-Cook ED, Orgeta V, Orrell M, Rees J, Russell IT (2012). REMCARE: reminiscence groups for people with dementia and their family caregivers - effectiveness and cost-effectiveness pragmatic multicentre randomised trial. Health Technol Assess.

[5] Daly L, McCarron M, Higgins A, McCallion P (2013). 'Sustaining Place' - a grounded theory of how informal carers of people with dementia manage alterations to relationships within their social worlds. J Clin Nurs.

[6] Dassel KB, Carr DC, Vitaliano P (2017). Does Caring for a Spouse With Dementia Accelerate Cognitive Decline? Findings From the Health and Retirement Study. Gerontologist.

[7] Hayes J, Zimmerman MK, Boylstein C (2010). Responding to symptoms of Alzheimer's disease: husbands, wives, and the gendered dynamics of recognition and disclosure. Qual Health Res.

[8] (2017). World Health Organization.

[9] Gibson F (2011). Reminiscence and life story work: A practice guide.

[10] Subramaniam P, Woods B (2012). The impact of individual reminiscence therapy for people with dementia: systematic review. Expert Rev Neurother.

[11] Karlsson E, Sävenstedt S, Axelsson K, Zingmark K (2014). Stories about life narrated by people with Alzheimer's disease. J Adv Nurs.

[12] Lazar A, Thompson H, Demiris G (2014). A systematic review of the use of technology for reminiscence therapy. Health Educ Behav.

[13] Astell AJ, Ellis M, Bernardi L, Alm N, Dye R, Gowans G, Campbell J (2010). Using a touch screen computer to support relationships between people with dementia and caregivers. Interacting With Computers.

[14] Elfrink T, Juidema S, Kuinz M, Westerhof G (2017). The effectiveness of creating an online life story book on persons with early dementia and their informal caregivers: a protocol of a randomized controlled trial. BMC Geriatr.

[15] Hamel AV, Sims TL, Klassen D, Havey T, Gaugler JE (2016). Memory Matters: A Mixed-Methods Feasibility Study of a Mobile Aid to Stimulate Reminiscence in Individuals With Memory Loss. J Gerontol Nurs.

[16] Kerssens C, Kumar R, Adams AE, Knott CC, Matalenas L, Sanford JA, Rogers WA (2015). Personalized technology to support older adults with and without cognitive impairment living at home. Am J Alzheimers Dis Other Demen.

[17] Mulvenna M, Doyle L, Wright T, Zheng H, Topping P, Boyle K, Martin S (2011). Evaluation of card-based versus device-based reminiscing use photographic images. J Cyber Ther Rehab.

[18] Haesner M, Steinert A, O'Sullivan JL, Weichenberger M (2015). Evaluating an Online Cognitive Training Platform for Older Adults: User Experience and Implementation Requirements. J Gerontol Nurs.

[19] Bleakley CM, Charles D, Porter-Armstrong A, McNeill MDJ, McDonough SM, McCormack B (2015). Gaming for health: a systematic review of the physical and cognitive effects of interactive computer games in older adults. J Appl Gerontol.

[20] Delello JA, McWhorter RR (2015). Reducing the Digital Divide: Connecting Older Adults to iPad Technology. J Appl Gerontol.

[21] Regier NG, Gitlin LN (2018). Dementia-related restlessness: relationship to characteristics of persons with dementia and family caregivers. Int J Geriatr Psychiatry.

[22] Farina N, Page TE, Daley S, Brown A, Bowling A, Basset T, Livingston G, Knapp M, Murray J, Banerjee S (2017). Factors associated with the quality of life of family carers of people with dementia: A systematic review. Alzheimers Dement.

[23] McDonnell E, Ryan AA (2014). The experience of sons caring for a parent with dementia. Dementia (London).

[24] Melunsky N, Crellin N, Dudzinski E, Orrell M, Wenborn J, Poland F, Charlesworth G (2015). The experience of family carers attending a joint reminiscence group with people with dementia: A thematic analysis. Dementia.

[25] McHugh JE, Wherton JP, Prendergast DK, Lawlor BA (2012). Teleconferencing as a source of social support for older spousal caregivers: initial explorations and recommendations for future research. Am J Alzheimers Dis Other Demen.

[26] Archbold PG, Stewart BJ, Greenlick MR, Harvath T (1990). Mutuality and preparedness as predictors of caregiver role strain. Res Nurs Health.

[27] Bech P, Olsen LR, Kjoller M, Rasmussen NK (2003). Measuring well-being rather than the absence of distress symptoms: a comparison of the SF-36 Mental Health subscale and the WHO-Five Well-Being Scale. Int J Methods Psychiatr Res.

[28] Spruytte N, Van AC, Lammertyn F, Storms G (2002). The quality of the caregiving relationship in informal care for older adults with dementia and chronic psychiatric patients. Psychol Psychother.

[29] Gibson A, McCauley C, Mulvenna M, Ryan A, Laird EA, Bunting B, Ferry F, Bond R (2016). Assessing usability testing for people living with dementia. Rehab.

[30] Aydin M, Harmsen F, van SK, Stegwee R (2005). On the Adaptation of an agile Information systems development method. J Database Management.

[31] Mulvenna M, Gibson A, McCauley CO, Ryan AA, Bond R, Laird EA, Curran KJ, Bunting B, Ferry F (2017). Behavioural Usage Analysis of a Reminiscing App for People Living with Dementia and their Carers. Proceedings of the European Conference on Cognitive Ergonomics.

[32] Lewin K (1935). A dynamic theory of personality.

[33] Place S, Blanch-Hartigan D, Rubin C, Gorrostieta C, Mead C, Kane J, Marx BP, Feast J, Deckersbach T, Pentland AS, Nierenberg A, Azarbayejani A (2017). Behavioral Indicators on a Mobile Sensing Platform Predict Clinically Validated Psychiatric Symptoms of Mood and Anxiety Disorders. J Med Internet Res.

[34] Intille S, Rondoni J, Kukla C, Ancona I, Bao L (2003). A context-aware experience sampling tool. Proceedings of CHI 2003.

[35] Mulder I, Ter HG, Kort J (2005). SocioXensor: Measuring user behaviour and user experience in context with mobile devices,. Proceedings of Measuring Behavior.

[36] Wiebe DJ, Nance ML, Houseknecht E, Grady MF, Otto N, Sandsmark DK, Master CL (2016). Ecologic Momentary Assessment to Accomplish Real-Time Capture of Symptom Progression and the Physical and Cognitive Activities of Patients Daily Following Concussion. JAMA Pediatr.

[37] Kingston D, Janes-Kelley S, Tyrrell J, Clark L, Hamza D, Holmes P, Parkes C, Moyo N, McDonald S, Austin M (2015). An integrated web-based mental health intervention of assessment-referral-care to reduce stress, anxiety, and depression in hospitalized pregnant women with medically high-risk pregnancies: a feasibility study protocol of hospital-based implementation. JMIR Res Protoc.

[38] Cook N, Winkler SL (2016). Acceptance, Usability and Health Applications of Virtual Worlds by Older Adults: A Feasibility Study. JMIR Res Protoc.

[39] Toscos T, Daley C, Heral L, Doshi R, Chen Y, Eckert GJ, Plant RL, Mirro MJ (2016). Impact of electronic personal health record use on engagement and intermediate health outcomes among cardiac patients: a quasi-experimental study. J Am Med Inform Assoc.

[40] Webster JD, Bohlmeijer ET, Westerhof GJ (2010). Mapping the Future of Reminiscence: A Conceptual Guide for Research and Practice. Res Aging.

[41] Kordelaar K van, Vlak A, Kuin Y, Westerhof G (2007). Een handleiding en werkboek voor intergenerationele gesprekken [Time Traveling: A manual for intergenerational groups]. Nijmegen: Centre for Psychogerontology.

[42] Des JDC, Lyles C, Crepaz N (2004). Improving the reporting quality of nonrandomized evaluations of behavioral and public health interventions: the TREND statement. Am J Public Health.

[43] Sim J, Lewis M (2012). The size of a pilot study for a clinical trial should be calculated in relation to considerations of precision and efficiency. J Clin Epidemiology.

[44] Lyons KS, Sayer AG, Archbold PG, Hornbrook MC, Stewart BJ (2007). The enduring and contextual effects of physical health and depression on care-dyad mutuality. Res Nurs Health.

[45] Tetz KB, Archbold PG, Stewart BJ, Messecar D, Hornbrook MC, Lucas SA (2006). How frail elders evaluate their caregiver's role enactment: a scale to measure affection, skill, and attentiveness. J Fam Nurs.

[46] Schumacher KL, Stewart BJ, Archbold PG (2007). Mutuality and preparedness moderate the effects of caregiving demand on cancer family caregiver outcomes. Nurs Res.

[47] (1998). World Health Organization.

[48] Woods RT, Orrell M, Bruce E, Edwards RT, Hoare Z, Hounsome B, Keady J, Moniz-Cook E, Orgeta V, Rees J, Russell I (2016). REMCARE: Pragmatic Multi-Centre Randomised Trial of Reminiscence Groups for People with Dementia and their Family Carers: Effectiveness and Economic Analysis. PLoS One.

[49] Henkel V, Mergl R, Kohnen R, Allgaier A, Möller H, Hegerl U (2004). Use of brief depression screening tools in primary care: consideration of heterogeneity in performance in different patient groups. Gen Hosp Psychiatry.

[50] Liwowsky I, Kramer D, Mergl R, Bramesfeld A, Allgaier A, Pöppel E, Hegerl U (2009). Screening for depression in the older long-term unemployed. Soc Psychiatry Psychiatr Epidemiol.

[51] de WM, Pouwer F, Gemke RJBJ, Delemarre-van DWHA, Snoek FJ (2007). Validation of the WHO-5 Well-Being Index in adolescents with type 1 diabetes. Diabetes Care.

[52] Löwe B, Spitzer RL, Gräfe K, Kroenke K, Quenter A, Zipfel S, Buchholz C, Witte S, Herzog W (2004). Comparative validity of three screening questionnaires for DSM-IV depressive disorders and physicians' diagnoses. J Affect Disord.

[53] Topp CW, Østergaard SD, Søndergaard S, Bech P (2015). The WHO-5 Well-Being Index: a systematic review of the literature. Psychother Psychosom.

[54] IBM (2016). IBM SPSS Statistics for Windows, Version 23.0.

[55] Bannon W (2015). Missing data within a quantitative research study: How to assess it, treat it, and why you should care. J Am Assoc Nurse Pract.

[56] Pallant J (2010). SPSS Survival Manual.

[57] Folstein MF, Folstein SE, McHugh PR (1975). “Mini-mental state”. A practical method for grading the cognitive state of patients for the clinician. J Psychiatr Res.

[58] Sheikh J, Yesavage J (1986). Geriatric Depression Scale (GDS): Recent evidence and development of a shorter version. Clin Gerontol.

[59] Kerkhof YJF, Graff MJL, Bergsma A, de VHHM, Dröes R (2016). Better self-management and meaningful activities thanks to tablets? Development of a person-centered program to support people with mild dementia and their carers through use of hand-held touch screen devices. Int Psychogeriatr.

[60] Krebs P, Duncan DT (2015). Health App Use Among US Mobile Phone Owners: A National Survey. JMIR Mhealth Uhealth.

[61] Becker S, Miron-Shatz T, Schumacher N, Krocza J, Diamantidis C, Albrecht U (2014). mHealth 2.0: Experiences, Possibilities, and Perspectives. JMIR Mhealth Uhealth.

[62] Hale K, Capra S, Bauer J (2015). A Framework to Assist Health Professionals in Recommending High-Quality Apps for Supporting Chronic Disease Self-Management: Illustrative Assessment of Type 2 Diabetes Apps. JMIR Mhealth Uhealth.

[63] Alzheimer's Research UK (2015). Women and dementia: A marginalised majority.

